# Mapping the m1A, m5C, m6A and m7G methylation atlas in zebrafish brain under hypoxic conditions by MeRIP-seq

**DOI:** 10.1186/s12864-022-08350-w

**Published:** 2022-02-08

**Authors:** Wei Li, Xiaoyu Li, Xunjie Ma, Wei Xiao, Jingjing Zhang

**Affiliations:** Affiliated Hospital of Guangdong Medical University & Key Laboratory of Zebrafish Model for Development and Disease of Guangdong Medical University, Zhanjiang, 524001 China

**Keywords:** RNA methylation, Zebrafish, Hypoxia, miRNA, Retroviruses

## Abstract

**Background:**

The epigenetic modifications play important regulatory roles in tissue development, maintenance of physiological functions and pathological process. RNA methylations, including newly identified m1A, m5C, m6A and m7G, are important epigenetic modifications. However, how these modifications are distributed in the transcriptome of vertebrate brains and whether their abundance is altered under pathological conditions are still poorly understood. In this study, we chose the model animal of zebrafish to conduct a systematic study to investigate the mRNA methylation atlas in the brain.

**Results:**

By performing unbiased analyses of the m1A, m5C, m6A and m7G methylation of mRNA, we found that within the whole brain transcriptome, with the increase of the gene expression levels, the overall level of each of these four modifications on the related genes was also progressively increased. Further bioinformatics analysis indicated that the zebrafish brain has an abundance of m1A modifications. In the hypoxia-treated zebrafish brains, the proportion of m1A is decreased, affecting the RNA splicing and zebrafish endogenous retroviruses.

**Conclusions:**

Our study presents the first comprehensive atlas of m1A, m5C, m6A and m7G in the epitranscriptome of the zebrafish brain and reveals the distribution of these modifications in mRNA under hypoxic conditions. These data provide an invaluable resource for further research on the involvement of m1A, m5C, m6A and m7G in the regulation of miRNA and repeat elements in vertebrates, and provide new thoughts to study the brain hypoxic injury on the aspect of epitranscriptome.

**Supplementary Information:**

The online version contains supplementary material available at 10.1186/s12864-022-08350-w.

## Introduction

In the 1950s, researchers discovered the first structurally modified nucleoside in yeast, namely, pseudouridine [1]. Subsequently, the N(6)-methyladenosine (m6A) modification of mammalian mRNAs was discovered in 1974 [2, 3]. However, due to the limitation of technology and the advanced structure of RNA, research progress on RNA epigenetic modification has been limited. Recently, with the prevalence of high-throughput technology development, a wave of research on epigenetic modification has been stimulated. The ubiquity and biological significance of the internal methylation of mRNA have been recognized [4, 5]. Therefore, the field of RNA epigenetic modification research has emerged.

Scientists have found that m6A affects the ability of some proteins to bind to RNA, such as heterogeneous nuclear ribonucleoprotein C (HNRNPC) [6]. m6A also affects many biological processes, such as pre-mRNA processing, protein translation initiation and mRNA degradation [7, 8]. Based on the above m6A functions at the molecular level, it also affects biological processes, such as development, metabolic diseases, sex differentiation and tumorigenesis [9–12]. Most importantly, the m6A demethylase FTO was discovered [4], paving the way for increased research on RNA epigenetic modification and establishing a foundation for the study of other RNA methylation modifications. It has been demonstrated that the abundance of m6A ranges from 0.1 - 0.4% of the total adenosine residues in cellular mRNA [13]. With the development of technology, researchers have also found m1A, m5C, m7G and other modifications of mRNA. The abundance of m1A on mRNA ranges from 0.015 - 0.054% in mammalian cells and 0.16% in mammalian tissues [14, 15]. Approximately 0.4% of human cells are modified by m5C [16], and the abundance of m7G in mammalian mRNA ranges from approximately 0.02 - 0.05% (excluding the m7G cap structure) [17]. However, it is still unknown whether hypoxia could affect the abundance of methylation modifications on mRNAs, which further affects the expression levels of these genes.

Hypoxia is a significant feature of the solid tumor microenvironment and it has been proved that the m6A modification plays a vital role in regulating tumor development in hypoxic microenvironment [18–20]. Moreover, brain is also very sensitive to and is the primary target of global hypoxia, such as in the patients of ischemic strokes and in the embryos suffering *perinatal hypoxia.* Whether the effects of the hypoxic microenvironment on the brain in the perinatal period and on solid tumors are affected by m1A, m5C or m7G modifications is still poorly understood. To better study the role of RNA methylation in brain tissue under a hypoxic microenvironment, we used a vertebrate model animal of zebrafish to conduct a systematic study.

Epigenetic mechanisms, including DNA methylation, histone modification, noncoding RNA and chromatin remodeling, have been demonstrated being involved in perinatal brain injuries [21, 22]. The m6A is rapidly and transiently modified in the RNA region corresponding to DNA damage, and this modification occurs on the polyA tails of many transcripts [23]. Studies also showed that mRNA is modified by m5C at the site of DNA damage. Because the RNA methyltransferase TRDMT1 is recruited to DNA damage sites, it promotes the generation of m5C and thus regulates homologous recombination, suggesting that post-transcriptional modifications of RNA can also be used as DNA damage codes to affect DNA repair [24]. These suggest that RNA base modification might correlate with DNA damage. In addition, m6A exhibits a dynamic response to external stimuli, which changes the expression pattern of the body to resist and adapt to stimulation [25–28]. m1A and m7G modifications on mRNAs have also been reported to respond to external stimuli, such as heat stress and glucose starvation [15, 29]. These results indicate that some methylation modifications on RNA are dynamic and reversible. In the animal brain, the abundance of m1A and m6A modifications on mRNA are the highest [15, 30]. Moreover, m6A has been extensively proven to function on brain development and diseases.

Endogenous retroviruses (ERVs), identified in human brain cells and mouse models, are revealed being associated with many central nervous system diseases [31–33]. Recently, it is reported that ERVs could be affected by m6A and function on maintaining cell integrity and regulating gene expression [34, 35]. These findings provide a good basis to study whether ERVs are also affected by RNA methylation in the brain.

Although RNA methylation has been reported playing vital roles in embryonic development and in the pathological processes, e.g., in the brain suffering ischemic and hemorrhagic stroke, no systematic mapping of the methylation atlas in brain tissue has been conducted till now. How these RNA methylations are altered under these pathological conditions is still unclear. In this study, the model animal of zebrafish was applied for this comprehensive and systematic study. The mRNA methylation atlas indicates an important role of RNA methylation in maintaining the cerebral physiological functions. It also provides a comprehensive database of epitranscriptome marks present in adult brain, which may help to reveal potential biomarkers on epigenetic levels in some cerebral diseases related to hypoxic injury.

## Results

### Overview of m1A, m5C, m6A and m7G methylation abundance in zebrafish brain under hypoxic conditions

**Fig. 1:**
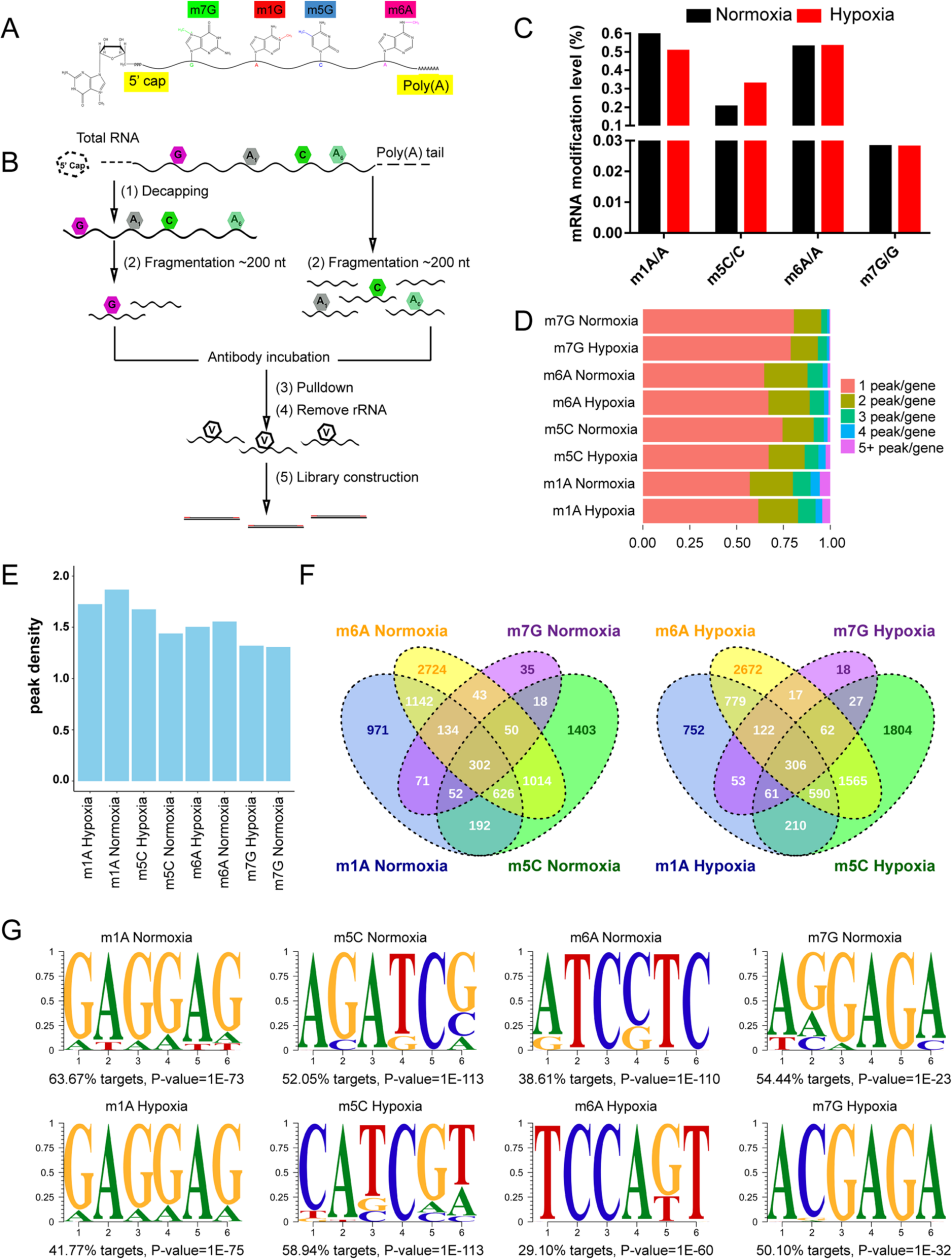
MeRIP-seq mapped transcriptome-wide distributions of m1A, m5C, m6A and m7G sites in the zebrafish brain under hypoxic conditions. **A** Schematic diagram of base modifications (m1A, m5C, m6A and m7G) within mRNA. **B** Schematic outline of MeRIP-seq. RNA was extracted from zebrafish brain tissue, and mRNA was enriched by PolyA, chemically fragmented to approximately 200 nt, and incubated with anti-m1A, m5C, m6A and m7G antibodies or IgG. Then, the target mRNA fragment was pulled down, and library construction was performed by high-throughput sequencing. **C** The position information of all the methylation regions on the mRNA was obtained by Bedtools GetFasta, the number of bases in the peak section was counted in each sample, and the proportion of methylation modifications within mRNA was calculated. These m1A, m5C, m6A and m7G of internal mRNA proportions were found to be 0.6203, 0.2011, 0.5264 and 0.0281% in the normal oxygen group and 0.5029, 0.3258%, 0.5294 and 0.0279% in the hypoxia group, respectively. **D** The percentage of methylated genes with 1, 2, 3, 4, or 5+ peaks per gene in the indicated zebrafish brain tissues, of which 1 peak gene accounted for the most. **E** The average peak density of all methylated genes in zebrafish brain tissue. **F** Venn diagram of methylated genes with m1A, m5C, m6A and m7G. The number of four modified genes in the normal oxygen group was 302, while that in the hypoxia group was 306. **G** Motifs of m1A, m5C, m6A and m7G. m1A and m7G tended to be GA-rich under both hypoxic and normoxic conditions, while m5C and m6A seem to be a little difference

Recent studies have revealed a variety of internal modifications of eukaryotic mRNA, including additional methylations of adenosine to form N(1)-methyladenosine (m1A), C(5)-methylcytosine (m5C), N(6)-methyladenosine(m6A), and N(7)-methylguanosine (m7G) [13, 15, 17, 36, 37]. To investigate the mRNA methylation atlas in zebrafish brain, the brains from adult fish were collected for MeRIP-Seq following published protocol (Fig. 1A & B) [13]. Meanwhile, to reveal the alterations of the methylated modifications of mRNA in the brain transcriptome post-hypoxia induction, zebrafish were first incubated in a hypoxia-preconditioned tank until it reaches the physiological status of hypoxia [38]. Then, the brains from normoxia- or hypoxia-treated zebrafish were extracted for RNA-Seq and MeRIP-Seq respectively. The sequencing results indicated that all the four RNA methylation modifications of m1A, m5C, m6A and m7G are widely distributed within mRNAs of zebrafish brain under both hypoxic and normoxic conditions (Fig. 1C).

Based on the high-throughput sequencing results, we obtained the proportion of methylated modified bases to the total number of bases by bioinformatics analysis as follows: m1A/A (≈0.6%), m5C/C (≈0.2%), m6A/A (≈0.5%) and m7G/G (≈0.02%). Importantly, we found relatively high m1A/A and m6A/A levels in the zebrafish brain (Fig. 1C), which is consistent with their abundance in the mammalian brain [13, 15, 25], indicating that RNA methylation in the brain is partly conserved between zebrafish and mammals. We found that the ratio of m1A/A was decreased to approximately 0.5% and that the ratio of m5C/C was increased to approximately 0.3% after exposure to hypoxic conditions, but the ratio of m6A/A and m7G/G showed little change (Fig. 1C). These results provide a clear overview of the abundance of methylation in zebrafish brain tissue under hypoxic and normoxic conditions. Based on MeRIP-seq, we found that more than 50% of the methylated transcripts were methylated once (Fig. 1D), and each methylated transcript carries approximately 1.5 methylation peaks on average (Fig. 1E). Under normoxic conditions, we identified 3948, 4930, 7311 and 877 potential genes with m1A, m5C, m6A and m7G sites, respectively, within the mRNA. We found that the number of m1A peaks per m1A-methylated gene in the hypoxia group was reduced compared with that in the normoxia group, while the number of m5C peaks per m5c-methylated gene in the hypoxia group was increased compared with that in the normoxia group (Table 1). Subsequently, we found 302 and 306 methylated transcripts in the normoxia and hypoxia groups, respectively, indicating that the four methylated transcripts were almost not affected under hypoxic conditions (Fig. 1F). All the identified methylated nucleotides for each gene’s mRNA are listed in Supplemental Table 1, with the information of gene name, gene ID and transcript ID. Next, to test whether these four methylation peaks in zebrafish brain tissue were related to potential sequence elements, we performed an unbiased motif search using HOMER software. We found that both the m1A and m7G mRNA peaks tended to have GA-rich motifs under both hypoxic and normoxic conditions, but there were some differences in the m5C and m6A motifs between the hypoxia and normoxia groups (Fig. 1G).

**Table 1:** Detailed information of m1A, m5C, m6A and m7G genes with 1, 2, 3, 4, or 5+ peaks per gene in the zebrafish brain under normoxia or hypoxia condition

	m1A		m5C		m6A		m7G	
	Normoxia	Hypoxia	Normoxia	Hypoxia	Normoxia	Hypoxia	Normoxia	Hypoxia
gene with 1 peak	2250 (56.99%)	2019 (61.61%)	3675 (74.54%)	4091 (67.16%)	4733 (64.74%)	4964 (67.04%)	707 (80.62%)	647 (78.90%)
gene with 2 peaks	909 (23.02%)	697 (21.27%)	816 (16.55%)	1169 (19.19%)	1692 (23.14%)	1622 (21.91%)	128(14.60%)	119 (14.51%)
gene with 3 peaks	373 (9.45%)	305 (9.31%)	267 (5.42%)	441 (7.24%)	592 (8.10%)	566 (7.64%)	27(3.08%)	39 (4.76%)
gene with 4 peaks	196 (4.96%)	118 (3.60%)	99 (2.01%)	237 (3.89%)	190 (2.60%)	174 (2.35%)	11(1.25%)	10 (1.22%)
gene with 5+ peaks	220 (5.57%)	138 (4.21%)	73 (1.48%)	153 (2.51%)	104 (1.42%)	78 (1.05%)	4(0.46%)	5 (0.61%)
genes in all	3948	3277	4930	6091	7311	7404	877	820

### Description of the characteristic distributions of m1A, m5C, m6A and m7G within mRNA

**Fig. 2:**
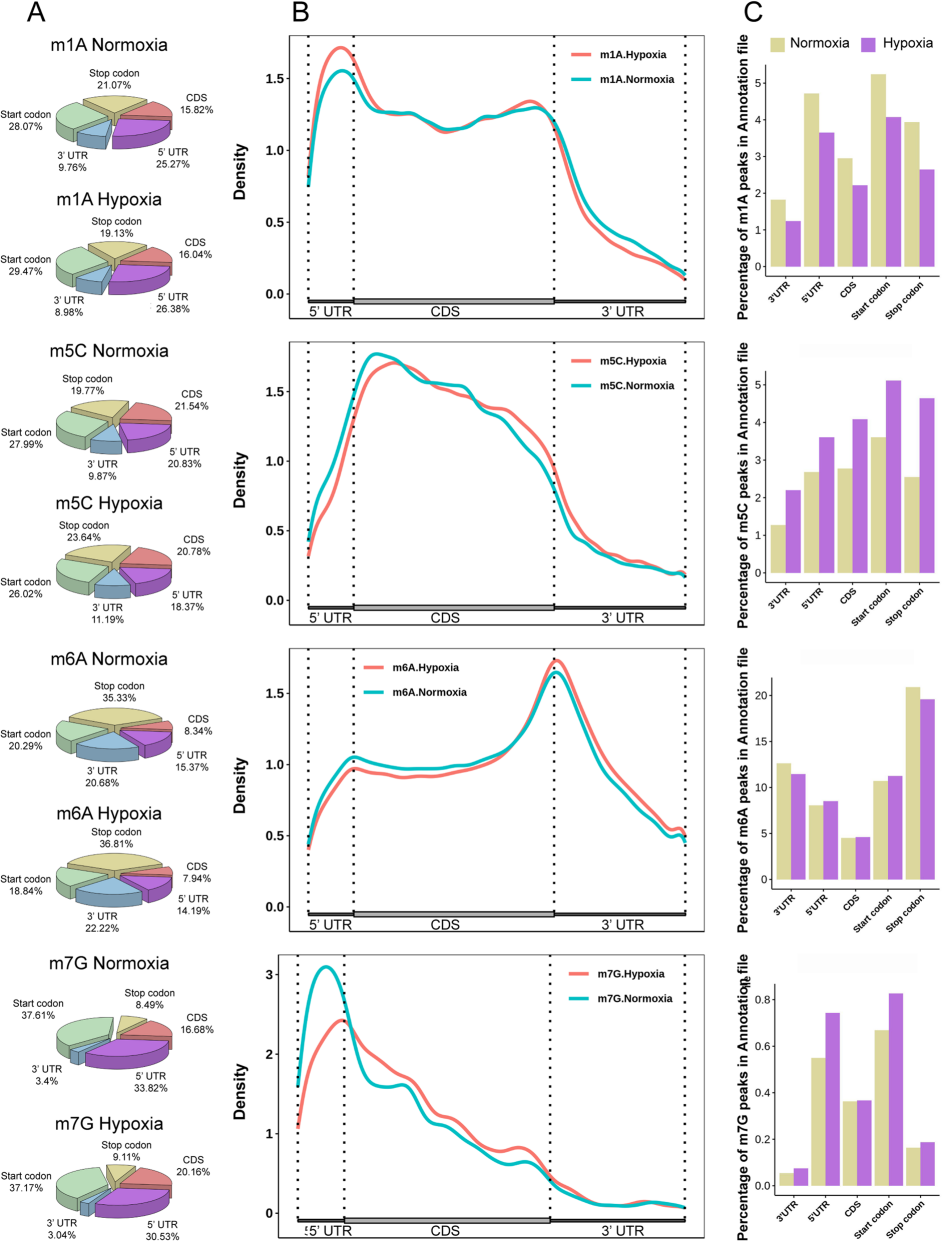
Distribution of m1A, m5C, m6A and m7G peaks across mRNA segments. **A** The pie chart shows the percentage of m1A, m5C, m6A and m7G sites in each non-overlapping segment. **B** Distribution of m1A, m5C, m6A and m7G sites across mRNA segments. Each segment was normalized according to its average length in Ref-seq annotation. **C** Describing the corresponding proportions of m1A, m5C, m6A and m7G methylated peaks in each region of RNA, where orange indicates the normal oxygen group and purple indicates the hypoxia group

To gain a better understanding of the distribution of m1A, m6A, m5C and m7G sites in zebrafish brain tissues, we analyzed the location of all four methylation sites with respect to the corresponding transcript features. These four methylation modification sites were detected in the three fragments of mRNA, 5’UTRs, coding sequences (CDSs) and 3’UTRs, and each methylated site exhibited its own distribution preference. We calculated the proportion of methylated peaks in the transcriptome and mapped the distribution of methylated peaks in the transcriptome. We found that m1A and m7G tended to be enriched in 5’UTRs and start codon regions, while m5C was enriched in CDS regions and m6A was enriched near the stop codon (Fig. 2A & B). Next, we counted the methylation peaks of each region of RNA and determined the peaks per region. Interestingly, we found that the abundance of m1A was downregulated in the hypoxic transcriptome, while that of m5C was upregulated in the hypoxic transcriptome, which was consistent with our results of the ratios of m1A/A and m5C/C in the zebrafish brain (Figs. 1C & 2C). Compared with that in the normoxia group, the abundance of m6A in each mRNA fragment in the hypoxia group was not different, but the abundance of m7G was downregulated in the 5’UTRs and start codon regions (Fig. 2C).

The above results clearly showed that the four methylation peaks exhibited significant differences in zebrafish brain tissue under hypoxic conditions. Further bioinformatics analysis showed that the corresponding peaks of m1A, m5C, m6A and m7G were 7373, 7138, 114,093 and 1150 in the normoxia group and 5656, 10,351, 11,163 and 1083 in the hypoxia group. We clearly found that the m1A peaks decreased and the m5C peaks increased in the hypoxic group, while the m6A and m7G peaks did not change (Supplemental Fig. 1A). We counted the number of differentially methylated peaks and found that the number of downregulated m1A and m7G peaks was 3.48 times and 3.13 times the number of upregulated peaks, respectively, while there was little difference between the two m5C and m6A modification peaks (Supplemental Fig. 1B). To examine the distribution of the four different methylation peaks within mRNA, namely, the up- and downregulated peaks, we found that the upregulated peaks of m1A were enriched in the stop codon regions, while the downregulated peaks were enriched in the start codon regions; the upregulated peaks of m5C were enriched in the stop codon regions, while the downregulated peaks were enriched in the CDS region; the upregulated peaks of m6A were enriched in the CDS regions, while the downregulated peaks were enriched in the stop codon regions; and the up- and downregulated peaks of m7G were enriched in the start codon regions (Supplemental Fig. 1C). We showed the distribution of m1A, m5C, m6A and m7G in the brain tissue transcriptome under hypoxic condition, which may provide clues for further study of the functions of these modifications in brain.

### Gene Ontology (GO) term enrichment analysis of differentially expressed gene categories, and the correlation analysis of the gene expression levels and RNA methylation levels

**Fig. 3:**
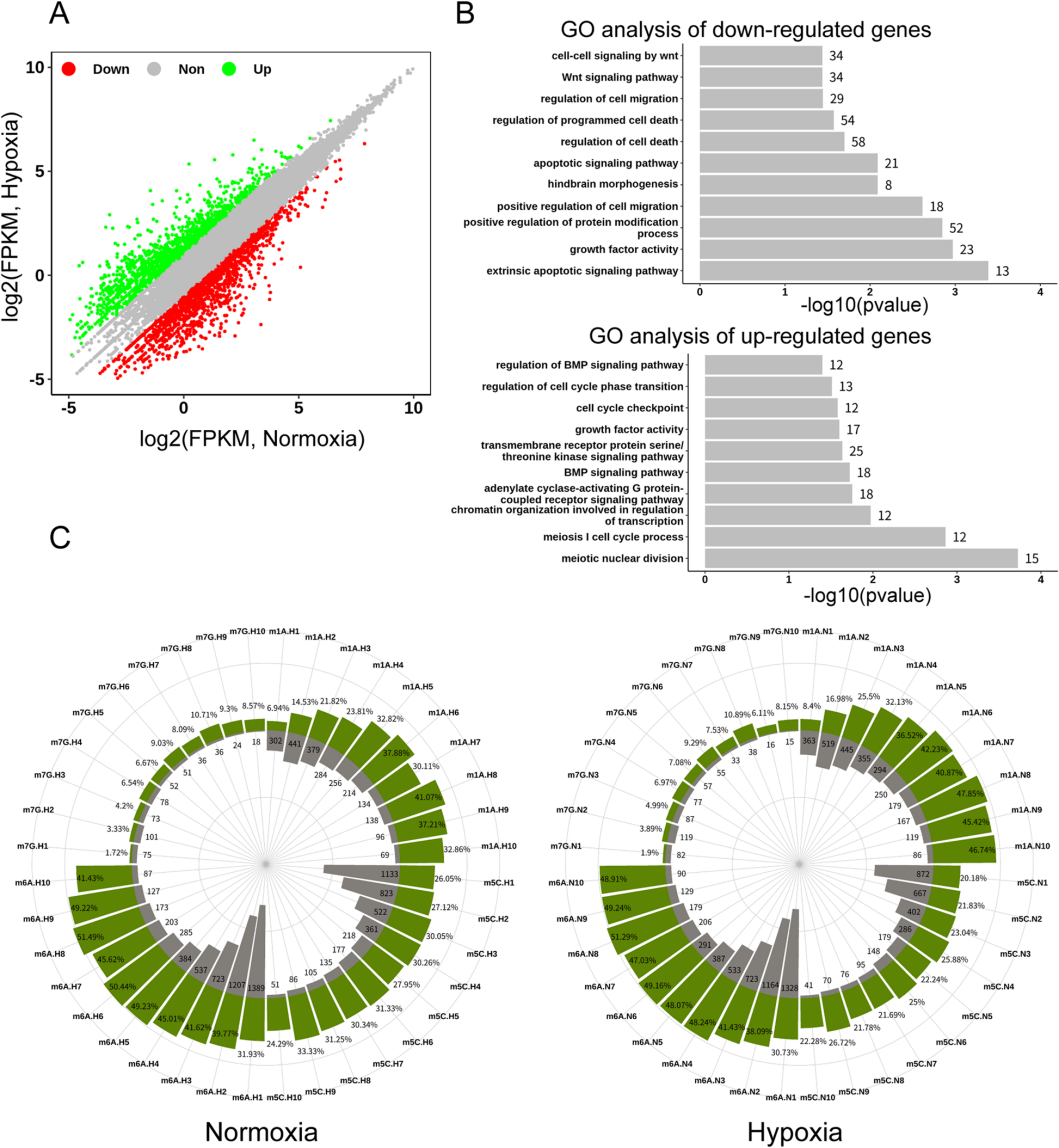
GO term enrichment analysis of differentially expressed gene categories and gene expression levels with the proportion of positively correlated methylated transcripts. **A** Through bioinformatics analysis, FC (fold change) was set to more than 2 times that of differentially expressed genes. The upregulated genes are represented by green and the downregulated ones by red; those with no difference are represented by gray. **B** GO term enrichment analysis revealed a distinct predominance of up/downregulated gene categories in zebrafish brain tissues. GO terms were analyzed with DAVID and further clustered using REVIGO. The ten most significantly enriched categories are shown. **C** The percentages of methylated genes among all genes (within one expression gearwheel) in zebrafish brain tissues accounted for a progressively larger fraction as the gene expression level increased (the expression level was equally divided into ten bins in a range of 1 ≤ FPKM ≤50). The gray gear indicates the gene expression level, and the green gear indicates the methylation level

Next, we aimed to understand the changes of the RNA expression levels after hypoxic treatment. To this end, RNA-seq was performed with the brain samples, and the results revealed that transcripts of brain tissue did change significantly after hypoxic induction. Based on the sequencing results, we identified a total of 3087 differentially expressed genes, including 1337 upregulated genes and 1750 downregulated genes (Supplemental Table 2), which were used in all subsequent analyses (Fig. 3A & Supplemental Fig. 2A). For the differentially expressed genes in the zebrafish brain tissues, we further clustered the up- and downregulated genes with REVIGO, and the 10 most abundant categories were listed as follows. We found that the downregulated genes were involved in cell invasion and apoptosis, while the upregulated genes were associated with biological events such as cell growth and the cell cycle (Fig. 3B). On the other hand, GO analysis of the downregulated genes revealed that the term of “hindbrain morphogenesis” related to the development of zebrafish brain was significantly enriched. Furthermore, we found that the expression of genes related to axon injury was altered, such as *socs3a* and *dpysl2b* (upregulated for 6-7 folds), and *neurog1* (downregulated for 4-5 folds) (Fig. 3B and Supplemental Fig. 2B) [39–41]. These results suggested that the brain injury may be related to the hypoxia induction.

We next analyzed the MeRIP-seq results to uncover the relationships between internal RNA methylation and transcriptional expression. The methylated genes were divided into an average of 10 expression bins (1 ≤ fragments per kilobase of exon model per million mapped fragments [FPKM] ≤ 50). When calculating the percentage of methylated genes relative to all the genes in each expression decile, the proportion of m1A-, m5C-, m6A- and m7G-methylated transcripts accounted for a progressively larger fraction with the increase in gene expression level in the zebrafish brain, and the same trend was observed in zebrafish brain tissue under hypoxic conditions (Fig. 3C and Supplemental Fig. 2C). Above results indicated that with the increase of the gene expression levels, the overall level of each of these four modifications on the related genes was also progressively increased (Fig. 3C and Supplemental Fig. 2C).

### Highly conserved internal m1A modification is correlated with the splicing of partial mRNA in zebrafish brain

**Fig. 4:**
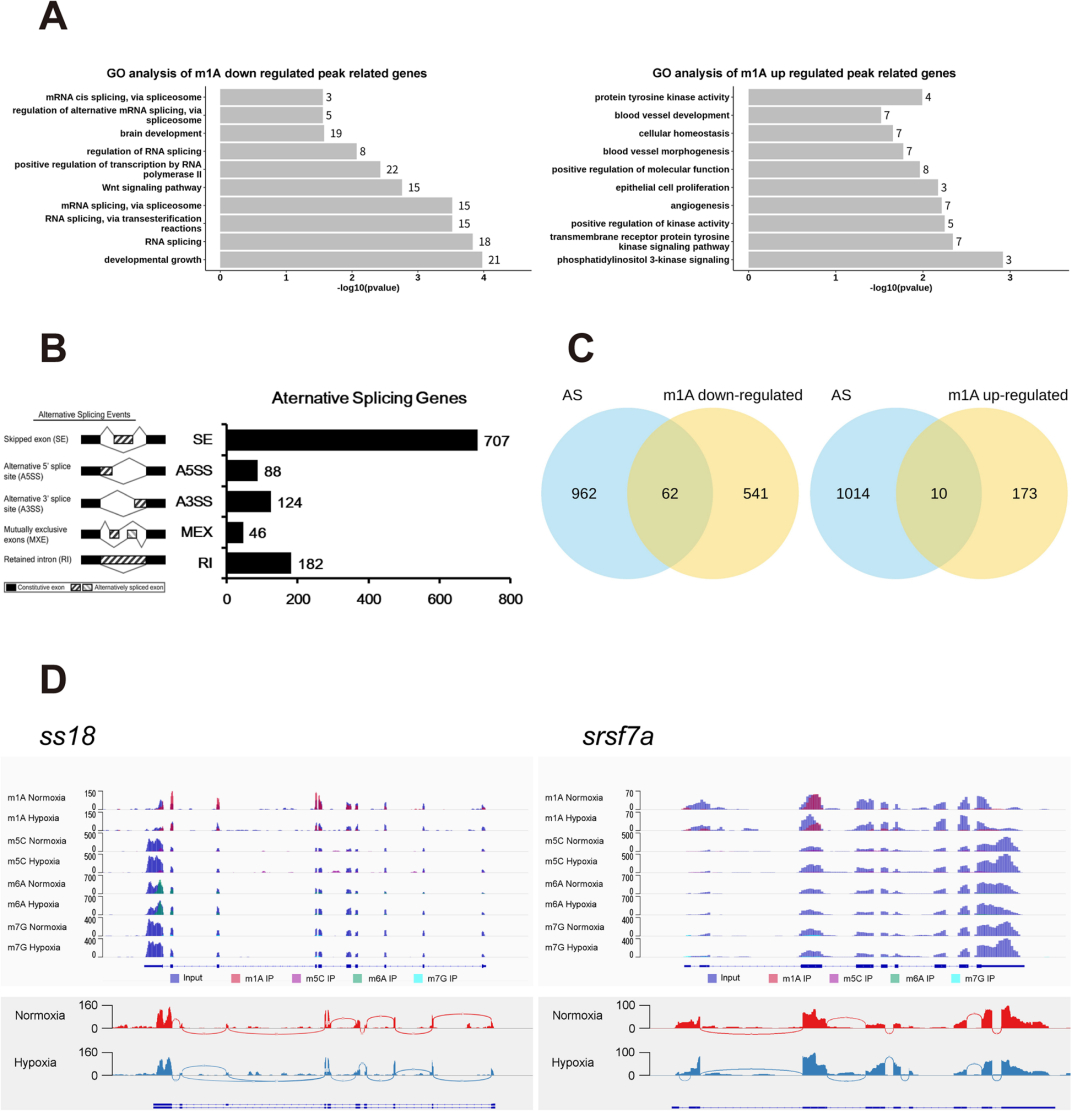
The conserved internal m1A modification is partially involved in mRNA splicing in the zebrafish brain. **A** Gene ontology (GO) analysis of internal m1A-methylated transcripts relative to all adequately expressed genes with FC enrichment greater than 2 times in zebrafish brain tissues (FPKM> 0). **B** rMats software was used to analyze RNA-seq data for RNA splicing events. The number of non-duplicated AS genes was counted (*P* < 0.05). Left: the pattern diagram of five AS events, right: the number of splicing genes in the form of a bar graph. **C** Venn diagram shows the intersection of differential m1A modifier genes and AS genes in zebrafish brain tissue. **D** AS genes with differential m1A modification were selected and visualized by IGV software. Top: blue represents input, other colors represent IP, and the vertical axis represents the coverage of sequencing. Bottom: visualization of alternatively spliced genes, in which the minimum splicing coverage is set to be greater than or equal to 10 (i.e., parameter: set junction coverage min: 10), left: *ss18*, right: *srsf7a*

Importantly, there is a wide distribution of m1A, m5C, m6A, and m7G modifications within mRNA, among which m1A is the most abundant in zebrafish brain tissue. This finding is consistent with the abundance of m1A modification in mammalian brain tissue (Fig. 1C) [15] and suggests that m1A may have certain conserved functions in vertebrate brains. To identify the m1A methylated sites within mRNAs based on m1A-MeRIP-seq, MACS software was utilized with default parameters. The differentially methylated sites were identified by diffReps. Bedtools is a fast, flexible toolset for genome arithmetic; we used this tool to annotate differentially methylated sites. Since multiple methylation sites may be annotated to the same gene, the annotated gene set first needs to be removed and duplicated and then applied for GO enrichment analysis. We found that upregulated genes were involved in kinase activity, angiogenesis and blood vessel morphogenesis and that downregulated genes were involved in variable RNA splicing, the Wnt signaling pathway and transcription activity, especially reflecting the importance of m1A to RNA splicing events (Fig. 4A). Alternative splicing (AS) is widespread in eukaryotic organisms. One study pointed out that 95% of genes containing multiple exons in the human genome have AS [42]. AS leads to transcript polymorphism, as an important transcriptional regulatory mechanism.

To explore the effect of m1A on AS of RNA, we performed splicing event analysis on the annotated genes in zebrafish brain tissue and found that exon skipping accounted for approximately 61.64% of the total events identified (Fig. 4B). It revealed that AS events occurred in 10.28% of m1A-downregulated genes, while only 5.78% of m1A-upregulated genes had AS, indicating that downregulated m1A-methylated genes were more likely to be targeted by AS (Fig. 4C). Next, we found that transcription activator *ss18* and splicing factor *srsf7* (*srsf7a*&*srsf7b*) presented low abundance of m1A modifications in the brains of hypoxic group, but there was no different in the peaks of m5C, m6A and m7G within *ss18*/*srsf7*. However, they did undergo AS, suggesting that m1A modification may participate in AS (Fig. 4D). Based on these findings, we speculated that zebrafish brain might respond to hypoxic induction by altering the level of m1A modification to regulate the expression of related genes.

### GO term enrichment analysis of differentially methylated genes

The number of methylated genes and differentially methylated genes were counted. We found that the number of m7G-methylated genes was the lowest, while the number of m6A-methylated genes was the highest (Supplemental Fig. 3A). According to our statistical analysis, the number of m1A- and m7G-downregulated genes was 3 times as high as that of the m1A- and m7G-upregulated genes (Supplemental Fig. 3B). To explore the possible biological functions of these differentially methylated genes, we performed GO term enrichment analyses of target mRNAs identified in the zebrafish brain under hypoxia. These differentially expressed genes were enriched in certain molecular and biological functions, which are described as follows. The m5C-downregulated genes were involved in cerebral angiogenesis and development, and the upregulated genes were involved in the development of the nervous system and cell growth; m6A-downregulated genes were involved in cell cycle and nervous system development, while the m6A-upregulated genes were involved in RNA transcription and MAP kinase activity; and m7G-downregulated genes were involved in cell adhesion and the notch signaling pathway, and the m7G-upregulated genes were mainly involved in cerebrovascular development and cell adhesion (Supplemental Fig. 3C). Since most of GO terms the methylated genes significantly enrich were closely related to brain angiogenesis or the development of the nervous system, it suggests a relationship among brain injury, RNA methylations and the hypoxia induction.

### Relationships among m1A, m5C, m6A, m7G and miRNA-binding sites within 3’UTRs

**Fig. 5:**
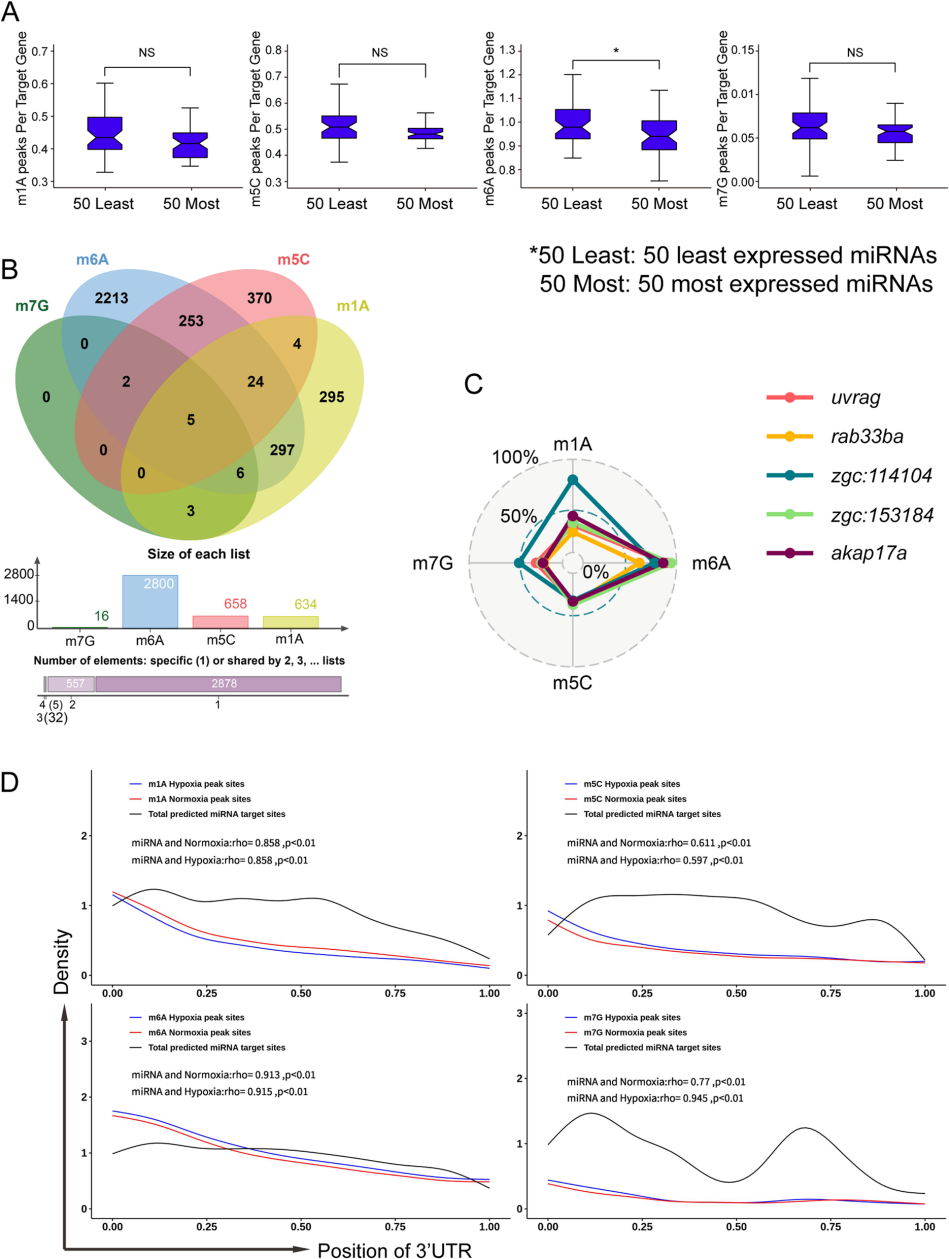
Relationship among m1A, m5C, m6A, m7G and miRNA-binding sites within 3’UTRs. **A** Association between 3’UTR methylation abundance and miRNA targeting. Methylation abundances of the targeted 3’UTRs for the 50 most/least abundant miRNAs in brain are shown using a bar chart, revealing a significantly greater percentage of m6A peaks within target mRNA 3‘UTRs for the 50 weakly expressed brain miRNAs compared with the 50 most abundant brain miRNAs (*p < 0.05, Wilcoxon test). The error bars indicate the highest and lowest values, and the box boundaries denote the first quartile, median, and third quartile (Public data: SRX685388, SRX685392). **B** (Upper) Venn diagram showing the overlap of genes comprising different kinds of methylation in their 3’UTR. (Middle) Bar chart displaying the number of M1a, m5C, m6A and m7G peaks within the 3’UTRs of methylation genes. (Lower) The number of 3’UTRs containing one, two, three or four kinds of methylation modification. **C** The enrichment scores of m1A, m5C, m6A and m7G of the five genes containing all four kinds of modifications are shown in the radar chart (*uvrag*: UV radiation resistance-associated gene; *rab33ba*: *rab3b*, member RAS oncogene family a; *akap17a*: A kinase (PRKA) anchor protein 17A). **D** Distribution of methylation peaks and miRNA target sites within 3’ UTRs. The frequency of methylation peaks under normoxia (red) or hypoxia (blue) and miRNA target sites (black) along the length of 3′ UTRs is shown. The relativity of methylation peak sites and miRNA target sites was calculated and displayed using the Pearson correlation coefficient

We next described the distribution of m1A, m5C, m6A and m7G peaks as well as the proportion of m1A, m5C, m6A and m7G in mRNA, and we found that they were all localized in the region of 3’UTR at certain proportions (Fig. 2A & B). Moreover, it has been reported that mammalian brain tissue has abundant miRNAs, which may be regulated by m6A [13, 43]. Therefore, we tried to analyze whether miRNA-targeted transcripts in zebrafish brain tissue were more likely to be methylated. We downloaded data from the NCBI (SRA) database and analyzed the expression of miRNAs in zebrafish brain tissue. We selected the 25 or 50 highest and lowest-expressed miRNAs, predicted their target sites along the 3’UTRs of transcripts identified by our RNA-seq data through TargetScan [44], and we analyzed whether these transcripts had a certain modification preference. The results showed that the 25 (50) miRNAs with the lowest abundant in the brain had a significantly greater percentage of m6A peaks within their target 3’UTRs than the 25 (50) miRNAs with the highest abundance (*p* < 0.05, Wilcoxon test) (Fig. 5A & Supplemental Fig. 4A).

Next, we sought to identify four posttranscriptional modification genes and miRNA-targeted regulatory genes. First, we analyzed all m1A, m5C, m6A and m7G peaks and used ChIPseeker (R package) [45] to annotate the target genes in the 3’UTR regions. We found that m6A, distributed in the 3’UTR of 2800 genes, was the most abundant form of methylation modification in the 3’UTR. The Venn diagram shows the intersection of four miRNA-targeted modified transcripts in the 3’UTR regions, and the results showed that the miRNA targeted 5 genes with m1A, m5C, m6A and m7G methylation modifications (Fig. 5B). Comparing the enrichment scores of the four modifications of the five genes through the radar chart, it was found that m6A had the highest abundance compared with the other modifications (Fig. 5C).

To further explore whether the preference of miRNA target selection is related to methylation modification, we predicted that miRNAs targeted transcripts with these four methylated modifications in the 3’UTRs, and the results showed that the miRNA targets were mainly enriched in the initial part of the 3’UTRs. Additionally, the enrichment showed a gradually decreasing trend, among which m6A-methylated 3’UTRs were the most apparent (Supplemental Fig. 4B). Subsequently, we used the Guitar package [46] to obtain the relative position of the miRNA target transcriptions, and these target genes exhibited RNA methylation modifications in the 3’UTRs. We calculated the relative position of the miRNA target sites, and combined with the peaks in the methylated 3’UTRs obtained from the Guitar package, a distribution curve was drawn and showed that the distribution of miRNA target positions positively correlated with those of the methylation peaks (Fig. 5D). In conclusion, we found that the four methylation modifications within mRNA (m1A, m5C, m6A and m7G) could have a relationship with the miRNA binding sites in the 3’UTR regions, but the relationship between m6A and miRNA was the most significant.

### Profile of the distribution of repeat elements in zebrafish brain tissue transcripts and the relationship with m1A, m5C, m6A and m7G RNA methylation

**Fig. 6:**
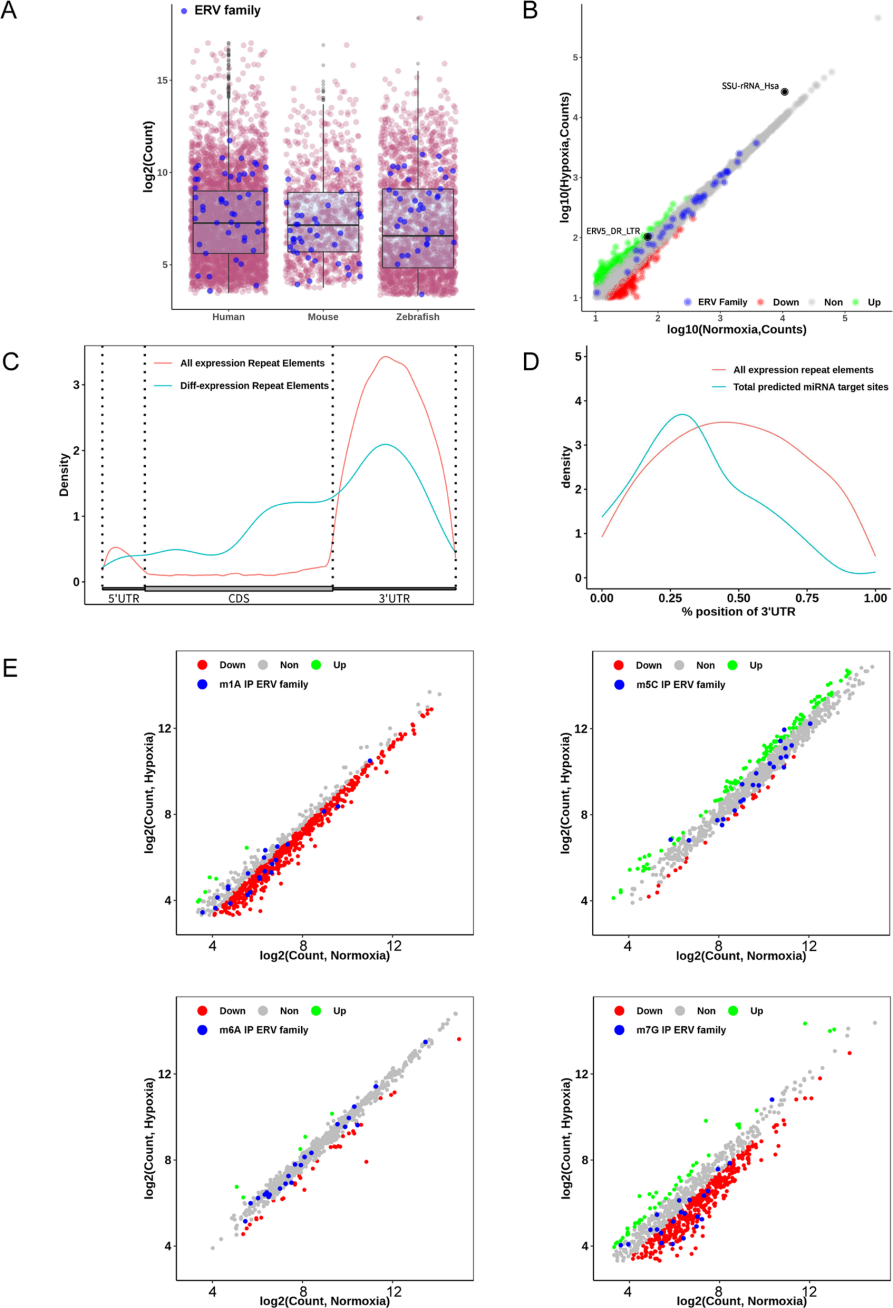
Profile of the distribution of repeat elements in zebrafish brain tissue transcripts and the relationship with RNA methylation of m1A, m5C, m6A and m7G. **A** RE expression levels in brain (human brain organoid) tissues of different species as determined by analyzing the corresponding RNA-seq data (RNA-seq data of human brain organoids and mouse brain tissue were downloaded from the GEO database with GSE171719 and GSE39911, respectively); purple and blue represent REs and ERV family members, respectively. **B** We analyzed the RE expression level (based on count) of RNA-seq data of zebrafish brain tissue (normoxia and hypoxia group) using featureCounts. After removing REs with counts less than 10 and setting 1.5 times the threshold, the differentially expressed REs were calculated and counted. **C** The distribution of all REs and differentially expressed REs on mRNA. The location information of REs and differentially expressed REs was extracted from the results of featureCounts, and the distribution curve was plotted based on the R package Guitar. **D** Correlation analysis between REs and miRNA targets of REs predicted by TargetScan. The distribution status of sites of REs and miRNA targets of REs in the 3’UTR was plotted through GuitarPlot. **E** Analysis of the expression level of methylation-modified REs. Four methylation (m1A, m5C, m6A and m7G) MeRIP-seq datasets were used to calculate the counts of REs using featureCounts software. After removing the REs with counts < 10 and normalizing the results to the logarithm of the expression of all REs, a plot of those four methylated REs was generated

Recently, it has been reported that miRNAs are derived from ERVs and other transposable elements [47–49]. For miRNA, our bioinformatics analysis found that the distribution of miRNA in zebrafish brain tissue was positively correlated with RNA methylation (Fig. 5 & Supplemental Fig. 4). Then we analyzed the expression of ERVs in vertebrate brain tissues on public data (human brain organoid: GSE171719 and mouse brain tissue: GSE39911), the result suggests that these ERVs in the zebrafish brain may also play an important role in brain development (Fig. 6A). However, the effect of hypoxia-induced zebrafish brain damage on repetitive elements, especially ERVs, is currently poorly understood. Our further analysis showed that there was no difference among the most of ERV family repeats under hypoxia treatment, but the ERV5_DR_LTR repeat has a relatively high expression trend (Fig. 6B). Furthermore, the maximum number of small-subunit rRNA (SSU rRNA) Hsa repeat element counts means the most obvious upregulation (Fig. 6B). Next, we described the distribution of repeat elements (REs) in the zebrafish brain transcript, and interestingly, we found that REs were enriched in 3’UTR regions, which was similar to the miRNA distribution (Fig. 6C). It has been reported that miRNAs can regulate the expression of ERVs and change cell fate [50]. As mentioned above, ERVs also produce miRNAs, so we described the relationship between the distribution of miRNAs and REs in the 3’UTR regions, and the results showed that they were positively correlated (Fig. 6D).

Interestingly, recent studies have shown that ERVs are regulated by m6A modification and has analyzed the chromatin modification of ERV genes, including H3K4me3, H3K27ac, and H3K9me3. The results confirmed that m6A mainly regulates ERVs expression through posttranscriptional modification [34, 35, 51], which raises an interesting question about the relationship between ERVs and RNA methylation in zebrafish brain tissue. Consequently, we analyzed MeRIP-seq data and found that most REs have a decreased level m1A- and m7G-methylation, especially in the ERV family, while the hypoxic did not appear to have a significant effect on m5C- and m6A-modification on repeating elements (Fig. 6E). These findings provide a new perspective for us to study the regulation of ERV by RNA methylation modification in vertebrate brain tissue and explore the influence of this regulation on the development of the nervous system and the occurrence and development of diseases.

### Distribution of repeat element transcripts in zebrafish genomes

When ERVs are integrated into the host genome, they can still replicate their own DNA and reinsert it into the host genome to regulate the gene expression network, and it has been reported that these sequences are enriched in the promoter regions of genes [52]. To analysis the distribution of repeat element transcripts in the zebrafish genomes, we first mapping the clean RNA-seq data back to the genomes available from Ensembl database. Then, the region information of REs along genomes was extracted based on the annotation file of repeat element acquired from Table Browser provided in the UCSC database by selecting the RepeatMasker track. By analyzing those regions position of REs, we found that they were mainly enriched in intergenic regions, introns and promoter regions (Supplemental Fig. 5A). Recent studies have reported that ERVs can be regarded as a new regulatory gene element or a new binding site as a transcription factor, and ERV gene products can even be reused for the benefit of the host [53]. Therefore, we analyzed the enrichment of all REs at transcription initiation sites (TSSs), and the results showed that REs were enriched at both ends of the TSS and were most widely distributed in the 10-100-kb region of the TSS (Supplemental Fig. 5B & C). The repeat element SSU-rRNA is upregulated the most in zebrafish brain tissue, but what is its distribution in the genome? Our analysis showed that SSU-rRNA was enriched in three segments of the genome (the gene region and 2 kb upstream and downstream of the gene) (Supplemental Fig. 5D). Through MeRIP-seq data analysis, we revealed that the four types of methylation were distributed in REs. The distribution of the result was quite different, and the simple description was as follows: m1A tended to be enriched downstream of the REs; m5C was extensively enriched in the REs and downstream; m6A was enriched at the end of the REs and downstream; and m7G only tended to be enriched at the end of the REs (Supplemental Fig. 5E). These studies indicate that REs are widely present in zebrafish brain tissues, providing new insight for investigating the potential interaction between RNA methylation and vertebrate REs.

## Discussion

Approximately 70% of the protein-coding genes between zebrafish and humans are conserved [54], therefore, zebrafish is an important model vertebrate in biomedical research and is extensively used to study human diseases and development. However, current epigenetic studies on zebrafish are mostly limited to embryonic development, and epigenetic studies on zebrafish brain tissue only involve DNA methylation. At present, there are few reports on RNA methylation in zebrafish brain tissue. Although it has been reported that brain tissue has abundant m1A and m6A modifications [15, 30], little is known about the distribution of m5C and m7G modifications within mRNAs in brain tissue. Indeed, the regulation of these modifications allows for unique multifunctionality at the level of gene expression. This regulation may involve the inherent dynamics of the epigenetic pathway, allowing the accumulation of m1A, m5C, m6A and m7G in response to a variable environment or as an aspect of programmed developmental change. In addition, m5C and m6A respond to DNA damage, indicating that they have a regulatory effect on organism damage and repair [23, 24]. Brain damage caused by hypoxic ischemic is common in the perinatal period, and this type of brain damage is known as hypoxic-ischemic encephalopathy (HIE) [55]. Hypoxia is a common feature of the tumor microenvironment that has been widely studied and confirmed, and it plays an important role in tumorigenesis and malignant development, serving as a key regulatory factor in tumor resistance to radiotherapy and chemotherapy and leading to poor prognosis. Besides in tumorigenesis, brain infractions in ischemic or hemorrhagic stroke are also a severe early consequence of hypoxic injury. Molecularly, hypoxia induces a large amount of DNA damage to tumor genomes, which affects the normal function of the DNA damage response pathway and ultimately leads to increased instability of tumor genomes. To explore whether the effects of hypoxia on brain injury were affected by m1A, m5C, m6A and m7G within mRNAs, we chose the zebrafish as a model animal to conduct a comprehensive analysis of the distribution of these four methylation modifications in brain tissue under hypoxia.

We found that zebrafish brain tissue had abundant m1A and m6A modifications (Fig. 1C), which is consistent with mammalian brain tissue, suggesting that these two modifications, the exact roles of which require further research, may play a vital role in the brain. In addition, the levels of m1A and m5C modification in brain tissue changed dramatically under hypoxic conditions, while the levels of m6A and m7G did not, indicating that m1A and m5C may be more sensitive to hypoxic stimuli (Fig. 1C & Supplemental Fig. 1A). We also found that the m1A modification level decreased in the hypoxia group, while the m5C modification level increased, so whether these two modifications have a certain relationship (antagonism) remains uncertain. In conclusion, these four methylation modifications in zebrafish brain tissue may potentially affect brain development and disease. Hypoxia-inducible factor (HIF-1) is a nuclear transcription factor that can be activated under hypoxic conditions and is a key mediator that specifically regulates oxygen homeostasis and is widely present in mammals. Hypoxia-inducible factors can activate the transcription factor p300 and bind to hypoxia response elements (HREs) to regulate the expression of multiple genes [56]. Therefore, whether this regulation is also affected by methylated modification under hypoxic conditions is also a question worthy of attention.

Recent studies have reported that the distribution of m6A methylation in mouse mRNA fragments is mainly concentrated in the vicinity of the 3’UTR and stop codons, and the distribution of m6A is highest among different kinds of mRNA methylation in mouse brain tissue [13]. It has been reported that the abundance of miRNA targets in the 3’UTR of the gene is significantly correlated with the abundance of m6A modifications, so we conducted a similar analysis and confirmed a significant correlation between these two factors but obtained results of opposite trends, which may be caused by species differences. We also investigated the distribution relationship between miRNA target abundance and modification abundance in the remaining three methylation-modified 3’UTRs, and significant positive correlations were found between the two distributions, strongly suggesting a relationship between RNA methylation and miRNA function. Although miRNAs can inhibit their target mRNA by promoting transcriptional degradation or translational inhibition, it is not clear whether the factors that determine which fate predominates are regulated by RNA methylation. Therefore, our data provide an idea for future research on miRNA and RNA methylation.

In recent studies, we found that zebrafish endogenous retrovirus (ZFERV) has an envelope protein gene that maintains a complete open reading frame. This protein is highly expressed in the thymus of zebrafish and has a certain degree of presence in other organizations [57, 58]. After exposure to hypoxia, the zebrafish repeat element expression regulation state, especially SSU-rRNA (Hsa), is changed. The mechanism underlying the upregulation of SSU-rRNA expression is unknown, and the role of SSU-rRNA in zebrafish brain tissue is still poorly understood. There is also abundant endogenous retrovirus in zebrafish brain tissue, and their expression status remains unchanged under hypoxic conditions. It has been reported that ERVs have enriched the diversity of the genome during species evolution; however, ERVs may also threaten individual safety by disrupting gene structure and expression. In mice, approximately 12% of pathological mutations are occurred by ERV integration; half of these mutations are affected by intracisternal A-particles (IAPs) of the ERVK family [59]. In humans, the expression of HERVs is involved in the regulation of cancer, infertility, aging and neurological disease [60]. The expression status of HERVs can be inhibited by DNA methylation and histone modification at the chromatin level, as well as by RNA modification and RNA interference at the posttranscriptional level, to inhibit the activity of HERVs. It is currently the most in-depth study of the HERV inhibition mechanism. Interestingly, it has recently been reported that m6A modification on RNA is involved in endogenous retroviral expression in embryonic stem cells, which in turn affects gene expression [34, 35, 51]. These results indicate that ERVs could be modified by m1A, m5C, m6A and m7G. Under hypoxic conditions, the modification levels of m1A and m7G on most ERVs were decreased, while the abundance of m5C and m6A seem to be unaffected. However, the mechanism and significance of the distribution patterns of m1A, m5C, m6A and m7G in the mRNA of ERVs need to be further analyzed. Further experimental studies are also needed to understand the exact nature of the relationship between zebrafish brain tissue REs and the four methylation modifications, to verify whether these observations are widely applicable to the development of other vertebrates, and to provide a reference for human brain development and disease research.

## Conclusions

In this study, we described the profiles of m1A, m5C, m6A and m7G in zebrafish brain tissue under hypoxic conditions. A large-scale transcriptome data set (RNA-seq and MeRIP-seq) was established, which can provide information about the expression status of RNA in zebrafish brain tissue and the modification of the epitranscriptome. These data will help with the reference studies of the regulatory effect of RNA methylation on brain injury, such as in stroke, and provide new strategies for clinical research.

## Materials and methods

### Preparation of samples

Adult AB line of wild-type zebrafish (*Danio rerio*) were raised in and obtained from the Key Laboratory of Zebrafish Model for Development and Disease of Guangdong Medical University according to standard methods [61]. Handling of zebrafish was performed in accordance with Guangdong State Regulations on Laboratory Animal Management (2010). For hypoxia treatment, oxygen in the water was firstly exhausted through the flowing of 8 L/min nitrogen until the dissolved oxygen value in the water was approximately 0.3-0.4 mg/L. Then adult male zebrafish per group were transferred into a 1 L chamber containing 800 mL pre-deoxidized water whose dissolved oxygen level was maintained by the continued flowing of the nitrogen (1 L/min). After approximately 5 min, zebrafish became motionless. Before the dissection of the brain tissue, both normoxic control and hypoxia-treated zebrafish were under anesthetic by immersion in 160 μg/mL *tricaine* (3-aminobenzoic acid ethyl ester; Sigma A-5040) solution for around 30 s till they did not respond to external stimuli. The anesthetized fishes were then put on ice for euthanasia and for dissection according to the standard protocol [61]. The extracted fresh brain tissues from both normoxia and hypoxia group were well preserved in liquid nitrogen for further RNA-Seq and MeRIP-Seq. For each normoxia control and hypoxia experimental group, 10 adult male zebrafish (3-4 month old) were chosen for the analyses. Three repeats of each experiment were performed and totally 30 fishes per group were collected. The brain tissues (around 0.02 g per brain) were then stored for further analysis in liquid nitrogen. For each analysis (m1A, m5C, m6A, m7G and RNA-Seq), the normoxia group and hypoxic group each consisted of 30 mixed brain tissues were analyzed parallelly by RNA-seq and MeRIP-Seq. In total, 300 adult fishes/brains were used for the whole study.

### RNA isolation, RNA-Seq and data analysis

Total RNA (1 μg) was treated to remove rRNAs using Ribo-Zero rRNA Removal Kits (Illumina, San Diego, CA, USA) following the manufacturer’s instructions. Next, RNA libraries were prepared by using the TruSeq Stranded Total RNA Library Prep Kit (Illumina, San Diego, CA, USA). Quality control was performed for the libraries with the BioAnalyzer 2100 system (Agilent Technologies, Inc., USA).10 pM denaturing libraries (single-strand DNA) were capture by the oligonucleotide sequence planted on the surface of Illumina flow cells. The clusters generated by in situ bridge-amplifying of the captured libraries were sequenced for 150 cycles on an NovaSeq 6000 sequencer.

Paired-end reads were harvested from an Illumina NovaSeq 6000 sequencer and were quality controlled by Q30. After 3′ adaptor trimming and low-quality read removal by Cutadapt software (v1.9.3), clean data was collected as input files of hisat2 (v2.0.4) which mapped the reads to the reference genome (GRCz11). Then, indexing by Ensembl gene annotation file (GRCz11.98.gtf), Cuffdiff software (part of cufflinks) was used to obtain the gene level FPKM as the expression profiles of mRNA. Differentially expressed genes was identified by calculating the fold change based on FPKM and further GO analysis of these differentially expressed genes was performed.

### MeRIP-Seq and data analysis

MeRIP-Seq was performed as follows according to the published procedure [13] with slight modifications. Briefly, RNA was disrupted with fragment buffer, and the resulting fragment length was approximately 200 nt. Subsequently, specific antibodies against m1A, m5C, m6A and m7G were used for immunoprecipitation was performed with the GenSeqTM RNA IP Kit (GenSeq Inc., China). Both the input sample without immunoprecipitation and the methylation IP samples were used for sequencing library generation with the NEBNext® Ultra II Directional RNA Library Prep Kit (New England Biolabs, Inc., USA). The libraries quality control was performed by using BioAnalyzer 2100 system (Agilent Technologies, Inc., USA). The sequencing processes were similar to the RNA-seq on a NovaSeq 6000 instrument.

The quality of raw data, directly harvested from the sequencer, is usually substandard and contains the sequencing adapter introduced in the library preparation step. Quality control is needed to make the data more reliable. Cutadapt (v1.9.3), command line software, was used to identify and trim 3′ adapter and low-quality bases. Subsequently, hisat2 (v2.0.4) was utilized to generated the position information by aligning clean data to the zebrafish reference genome (GRCz11). To identify the methylated sites on RNAs (peaks), MACS software was utilized. Differentially methylated sites were identified by diffReps. The motif predictions of MeRIP-Seq data were performed by utilizing a Perl script, findMotifGenome.pl, from HOMER software.

### Peak distribution

For MeRIP-Seq data, the sequences containing methylated sites (peak) were isolated from total RNA by a specific antibody. The location of those peaks affects many aspects of RNA. The peak distribution along RNA and accumulation in segments (5′ untranslated regions (UTRs), start codons, coding sequences (CDSs), stop codons, and 3’UTRs) of RNA were analyzed. For accumulation, peaks were annotated with the annotation file available in the Ensembl database by the intersectBed program from Bedtools software. The percentage of peaks located in each segment according to the annotation file was counted. For distribution, Guitar, an R package made by Xiaodong Cui et al. [46], was used. By utilizing the GuitarPlot function provided by Guitar, a plot of distribution features along mRNAs was generated when peak files called by MACS and an annotation file were provided.

### GO enrichment analysis

Gene expression was evaluated by FPKM. Before performing GO enrichment analysis, the gene set was prepared. For the RNA-Seq data, genes from all samples were filtered by FPKM > 0, and 2 folds was set to the threshold of the differential expression. The upregulated and downregulated genes were collected. For the MeRIP-Seq data, genes corresponding to differential methylation peaks were identified by diffReps software. Lists of differentially methylated peaks were prepared. GO enrichment analysis was performed for all gene lists with clusterProfiler, and the R packages were published in OMICS [62]. All of the significantly enriched GO items (*p* < 0.05) could be selected as candidates in this paper.

### Alternative splicing analysis

To investigate the events of AS during treatment with hypoxia in the zebrafish brain, rMats were used to identify all the differential AS events between the normoxia and hypoxia groups. The events for which *p* > 0.05 were generated by rMats were then removed, and the genes related to events for which *p* < 0.05 was calculated in five different alternative splice events included the skipping exon (SE), alternative 3′ splice site (A3SS), alternative 5′ splice site (A5SS), mutually exclusive exon (MXE) and retained intron (RI) [63]. For visualization of the methylated genes, the BAM file generated from MeRIP-seq data was first converted to a TDF file by IGV tools embedded in IGV. Then, TDF files were imported into IGV, and methylation peak regions and MeRIP-seq signals were visible when a gene symbol was input into the specified box. For splicing junctions, the BAM file from RNA-seq data was imported into IGV directly. The visualization of splice events was performed; however, a Sashimi plot is a good choice for understanding the junction coverage.

### The combined analysis with miRNA

The 3’UTR sequences of the methylated genes expressed in MeRIP-Seq were extracted from reference genome files (GRCz11.dna.toplevel.fa), and the miRNA expressed in zebrafish brain tissue was extracted from the NCBI Short Reads Archive with accession numbers of SRX685388 (https://www.ncbi.nlm.nih.gov/sra/?term=SRX685388) and SRX685392 (https://www.ncbi.nlm.nih.gov/sra/?term=SRX685392) [64]. Target sites of these specific miRNAs within the 3’UTRs of methylated genes under normoxic or hypoxic conditions were predicted through TargetScan60.pl, a Perl script provided by TargetScan (www.targetscan.org), and their relative coordinates within the 3’UTRs were calculated. GuitarPlot was used to depict the distribution of methylation peaks and miRNA target sites within 3’UTRs. Venn diagrams displaying the overlap of genes comprising different kinds of methylation within their 3’UTRs were produced using online worktools (http://jvenn.toulouse.inra.fr/app/index.html). A radar chart showing the enrichment scores of m1A, m5C, m6A and m7G of the five genes containing all four kinds of modifications was developed using the R package ggradar.

### The combined analysis with repeat elements

To study the REs in zebrafish brain tissue, we first evaluated their expression. An annotation file was downloaded from Table Browser provided by the UCSC database by selecting the RepeatMasker track. Specifically, the annotation messages were directly provided by the RepeatMasker database. For convenience, we used Table Browser to build the annotation file in GTF format. FeatureCounts, a subroutine of subread software, was used to calculate the count number of REs based on RNA-seq data and to annotate those REs by inputting the annotation file. The distribution of REs on mRNA was analyzed with Guitar. The analysis of REs distributed in the genome was performed with the ChIPseeker package [45]. To study the relationship of REs with methylation modification, we counted the methylated REs based on the MeRIP-seq data by featureCounts. The distribution of methylation sites on REs was further analyzed by deepTools. The regions of REs were extracted first based on the results of featureCounts using a custom R script. The BAM file of MeRIP-seq was converted to a BigWig file using the bamCoverage software provided by deepTools with default parameters to obtain coverage messages for the BAM file. Normalize RE regions and a coverage matrix were generated by computeMatrix, a source of deepTools, with the scale-region and “--binSize 50” parameters. To plot the distribution of methylation sites related to REs, plotProfile, software available from deepTools, was utilized with the necessary parameters. The Human and mouse brain RNA-Seq data was acquired from GEO dataset with accession numbers of GSE171719 (https://www.ncbi.nlm.nih.gov/geo/query/acc.cgi?acc=GSE171719) and GSE39911 (https://www.ncbi.nlm.nih.gov/geo/query/acc.cgi?acc=GSE39911).

### Statistical analysis

Significance levels (*p*-values) and sample sizes are provided in the text and figure legends or are indicated in the figures. Statistical analysis was performed using R. For measuring statistical significance, the Wilcoxon test or Pearson correlation analysis were used to calculate the p-value. *P*-values of < 0.05 were considered significant.

## Supplementary Information

**Additional file 1. Supplemental Table 1. **Detailed information of the methylated genes and the methylated nucleotides on the mRNAs.

**Additional file 2. Supplemental Table 2. **Differentially expressed genes analyzed in this study.

**Additional file 3. Supplemental Figure 1.** Distribution of differential methylation peaks of m1A, m5C, m6A and m7G in the transcriptome. . The fold enrichment of transcript methylation modification peaks analyzed by MACS2 software is displayed in 5 quantiles (boxplot), and the bottom of the box plot shows the number of methylation modification peaks in each group. B. The boxplot shows the up- and downregulated peaks (FC ≥ 2) for the four methylations, m1A, m5C, m6A and m7G. C. These four differential methylation peaks are profiled in different fragments of the transcript (red: downregulated methylation peaks, green: upregulated methylation peaks), and each region was found to have different degrees of enrichment, but each modification had its own preference. **Supplemental Figure 2.** Detection of the expression of all transcripts by RNA-seq and MeRIP-seq. A. The expression levels identified as unique to the brain were analyzed in zebrafish brain samples. The multilayer pie chart shows the number of differentially expressed genes, in which the number of upregulated genes is 1384 (blue) and the number of downregulated genes is 1801 (orange). B. The expression level of genes related to axon injury. C. The percentage of methylated genes in the normoxia group (top) and hypoxia group (bottom) in each expression bin are plotted. **Supplemental Figure 3.** GO term enrichment analysis of internal m5C, m6A and m7G-methylated transcripts relative to all adequately expressed genes. A. In the normal and hypoxia groups, the four methylation-modified genes (FPKM > 0) in the brain tissue of zebrafish were presented in a statistical histogram; the number of m6A genes was the highest, while the number of m7G genes was the lowest. B. Differentially methylation-modified (upregulation and downregulation FC ≥ 2, FPKM> 0) gene statistics histogram. C. Gene ontology enrichment bar chart of different gene categories in transcripts methylated in zebrafish brain tissues under hypoxic conditions, which shows the most significantly enriched category and phenotype-related entries. **Supplemental Figure 4.** The closest relationship between m6A and the miRNA-binding site in 3’UTRs. A. Association between 3’UTR methylation abundance and miRNA targeting. Methylation abundance of the targeted 3’UTR for the 25 most/least abundant miRNAs in brain are shown using a box-plot, revealing a significantly greater percentage of m6A peaks within target mRNA 3’UTRs for the 25 weakly expressed brain miRNAs compared with the 25 most abundant brain miRNAs (*p < 0.05, Wilcoxon test). B. Distribution of miRNA target sites along the 3′ UTRs of methylated genes. 3’UTRs are proportionally divided into ten parts, and the miRNA target abundance within each part is calculated and displayed using a bar chart. The relativity of miRNA target sites was calculated and displayed using the Pearson correlation coefficient. **Supplemental Figure 5.** Description of the distribution of repeat elements in zebrafish brain tissue genomes. A-C. Distribution of REs on the genome. The location information of REs was extracted from the featureCounts results, and ChIPseeker was used to analyze the distribution of these REs in the genome. A. The distribution of REs in each region of the genome. B. The distribution of REs in the range of 3 kb upstream and downstream of the TSS. C. The distribution percentage of REs within the scope of ±100 kb of the TSS. D. The distribution of SSU-rRNA on its corresponding genes. First, after extracting the location information of SSU-rRNA, bedtools was utilized to annotate those regions to the gene level, and the location information of its corresponding gene was obtained. Additionally all the SSU-rRNA sequences were extracted from the BAM file of RNA-seq, after which, deepTools was used to calculate and draw the SSU-rRNA distribution on these genes. E. The distribution of four methylation modification peaks on REs. To extract the location information of REs from the featureCounts results, deepTools was used to calculate the normalization matrix of MeRIP-seq and RE location information and to draw the distribution of methylation modification peaks on REs.

## Data Availability

The data that support the findings of this study have been deposited into NCBI Gene Expression Omnibus (GEO) database with the accession number of “GSE194284” (https://www.ncbi.nlm.nih.gov/geo/query/acc.cgi?acc=GSE194284). The Human and mouse brain RNA-Seq data was acquired from GEO dataset with accession numbers of “GSE171719” (https://www.ncbi.nlm.nih.gov/geo/query/acc.cgi?acc=GSE171719) and “GSE39911” (https://www.ncbi.nlm.nih.gov/geo/query/acc.cgi?acc=GSE39911), respectively. The miRNA-Seq data of zebrafish brain was obtained from the resource data bank of https://www.ncbi.nlm.nih.gov/sra/?term=SRX685388, https://www.ncbi.nlm.nih.gov/sra/?term=SRX685392.

## Abbreviations

m1A: N(1)-methyladenosine
m5C: C(5)-methylcytosine
m6A: N(6)-methyladenosine
m7G: N(7)-methylguanosine
ERVs: Endogenous Retrovirus
FPKM: Fragments Per Kilobase of exon model per Million mapped fragments
IGV: Integrative Genomics Viewer
REs: Repeat Elements
AS: Alternative Splicing
TSS: Transcription Start Sites
ZFERV: Zebrafish Endogenous Retrovirus
ERVK: Endogenous Retrovirus-K
HERV: Human Endogenous Retrovirus
