# Supplementary material for: Mapping the m1A, m5C, m6A and m7G methylation atlas in zebrafish brain under hypoxic conditions by MeRIP-seq
Source: BMC Genomics. 2022 Feb 8;23:105. doi: 10.1186/s12864-022-08350-w (PMC8822802; doi:10.1186/s12864-022-08350-w)
Supplement: Supplementary file 3 — Additional file 3. Supplemental Figure 1. Distribution of differential methylation peaks of m1A, m5C, m6A and m7G in the transcriptome. . The fold enrichment of transcript methylation modification peaks analyzed by MACS2 software is displayed in 5 quantiles (boxplot), and the bottom of the box plot shows the number of methylation modification peaks in each group. B. The boxplot shows the up- and downregulated peaks (FC ≥ 2) for the four methylations, m1A, m5C, m6A and m7G. C. These four differential methylation peaks are profiled in different fragments of the transcript (red: downregulated methylation peaks, green: upregulated methylation peaks), and each region was found to have different degrees of enrichment, but each modification had its own preference. Supplemental Figure 2. Detection of the expression of all transcripts by RNA-seq and MeRIP-seq. A. The expression levels identified as unique to the brain were analyzed in zebrafish brain samples. The multilayer pie chart shows the number of differentially expressed genes, in which the number of upregulated genes is 1384 (blue) and the number of downregulated genes is 1801 (orange). B. The expression level of genes related to axon injury. C. The percentage of methylated genes in the normoxia group (top) and hypoxia group (bottom) in each expression bin are plotted. Supplemental Figure 3. GO term enrichment analysis of internal m5C, m6A and m7G-methylated transcripts relative to all adequately expressed genes. A. In the normal and hypoxia groups, the four methylation-modified genes (FPKM > 0) in the brain tissue of zebrafish were presented in a statistical histogram; the number of m6A genes was the highest, while the number of m7G genes was the lowest. B. Differentially methylation-modified (upregulation and downregulation FC ≥ 2, FPKM> 0) gene statistics histogram. C. Gene ontology enrichment bar chart of different gene categories in transcripts methylated in zebrafish brain tissues under hypoxic conditions, whic [file 12864_2022_8350_MOESM3_ESM.docx]

**
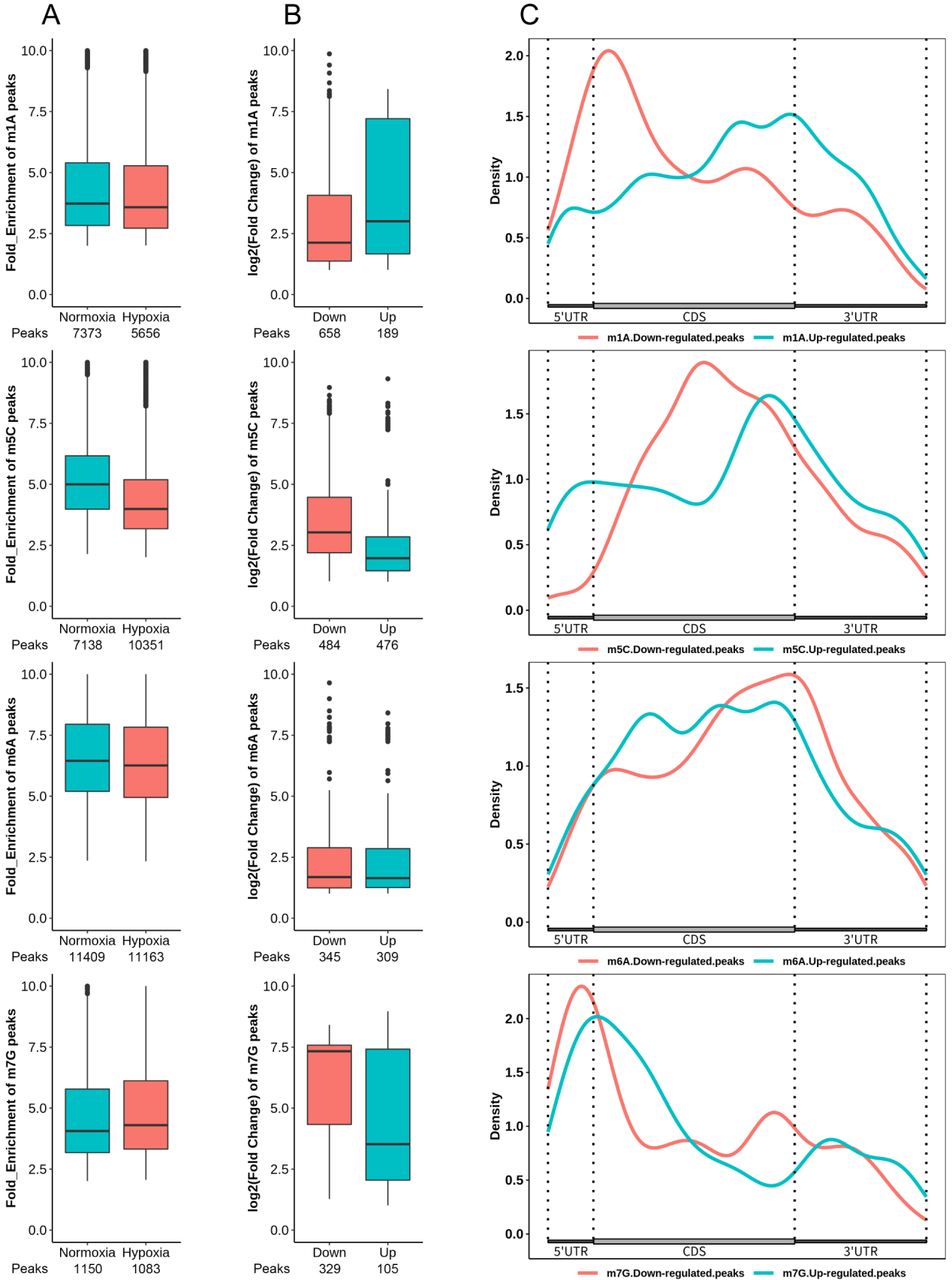
**

**Supplemental Figure 1. Distribution of differential methylation peaks of m1A, m5C, m6A and m7G in the transcriptome**

**A.** The fold enrichment of transcript methylation modification peaks analyzed by MACS2 software is displayed in 5 quantiles (boxplot), and the bottom of the box plot shows the number of methylation modification peaks in each group. **B.** The boxplot shows the up- and downregulated peaks (FC≥2) for the four methylations, m1A, m5C, m6A and m7G. **C.** These four differential methylation peaks are profiled in different fragments of the transcript (red: downregulated methylation peaks, green: upregulated methylation peaks), and each region was found to have different degrees of enrichment, but each modification had its own preference.


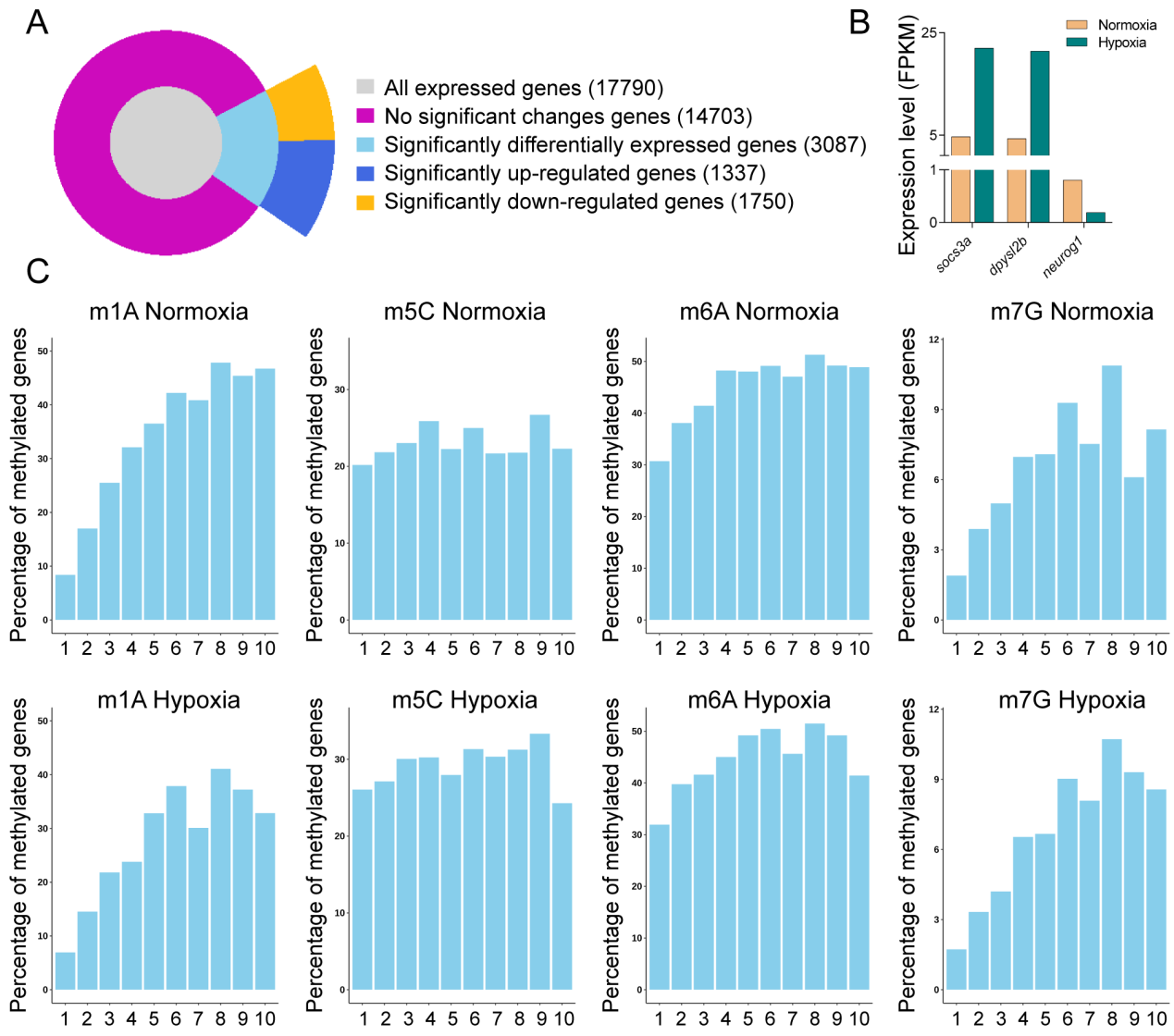


**Supplemental Figure 2. Detection of the expression of all transcripts by RNA-seq and MeRIP-seq**

**A.** The expression levels identified as unique to the brain were analyzed in zebrafish brain samples. The multilayer pie chart shows the number of differentially expressed genes, in which the number of upregulated genes is 1384 (blue) and the number of downregulated genes is 1801 (orange). **B.** The expression level of genes related to axon injury. **C**. The percentage of methylated genes in the normoxia group (top) and hypoxia group (bottom) in each expression bin are plotted.


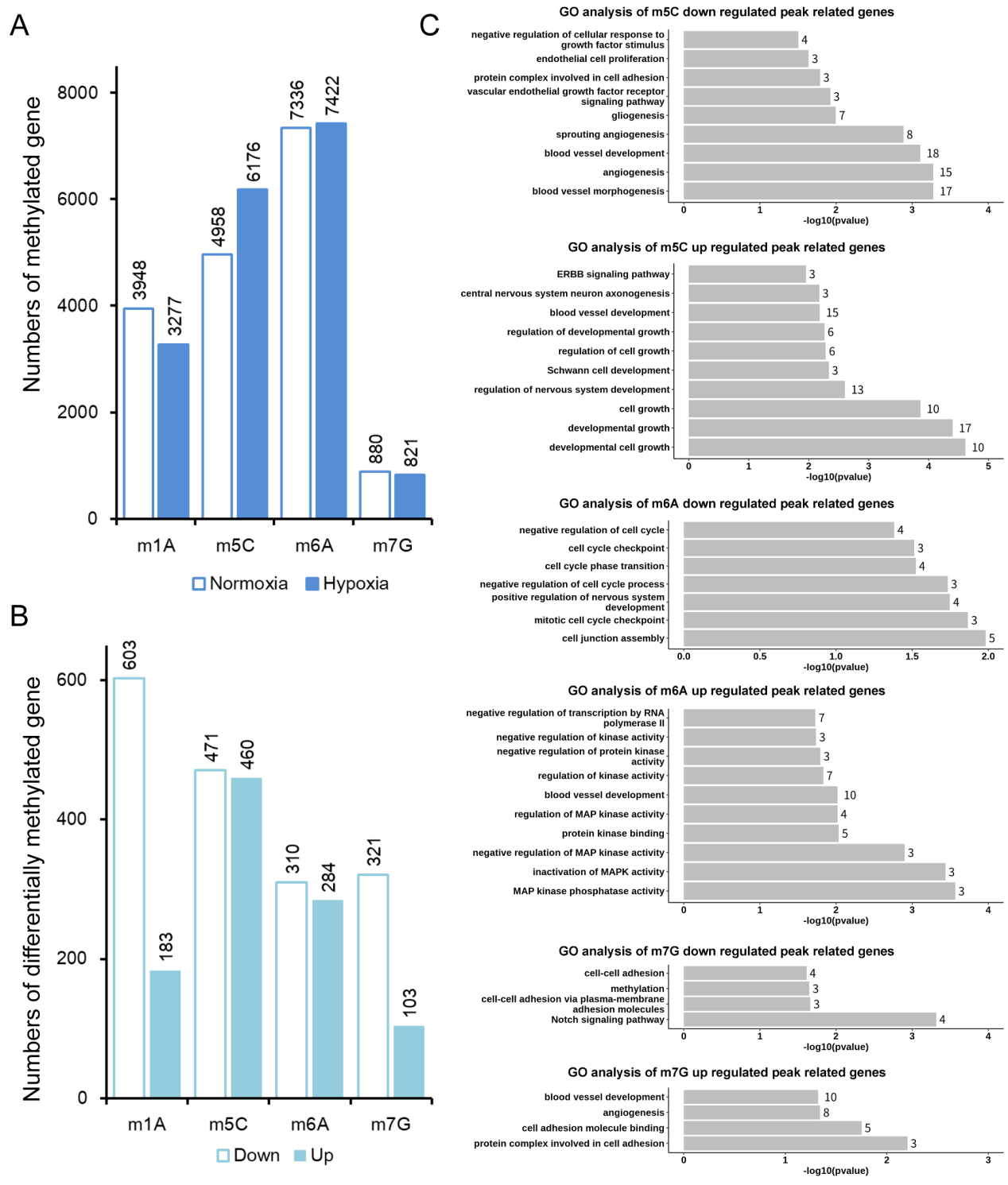


**Supplemental Figure 3. GO term enrichment analysis of internal m5C, m6A and m7G-methylated transcripts relative to all adequately expressed genes**

**A.** In the normal and hypoxia groups, the four methylation-modified genes (FPKM >0) in the brain tissue of zebrafish were presented in a statistical histogram; the number of m6A genes was the highest, while the number of m7G genes was the lowest. **B.** Differentially methylation-modified (upregulation and downregulation FC≥2, FPKM> 0) gene statistics histogram. **C.** Gene ontology enrichment bar chart of different gene categories in transcripts methylated in zebrafish brain tissues under hypoxic conditions, which shows the most significantly enriched category and phenotype-related entries.

**
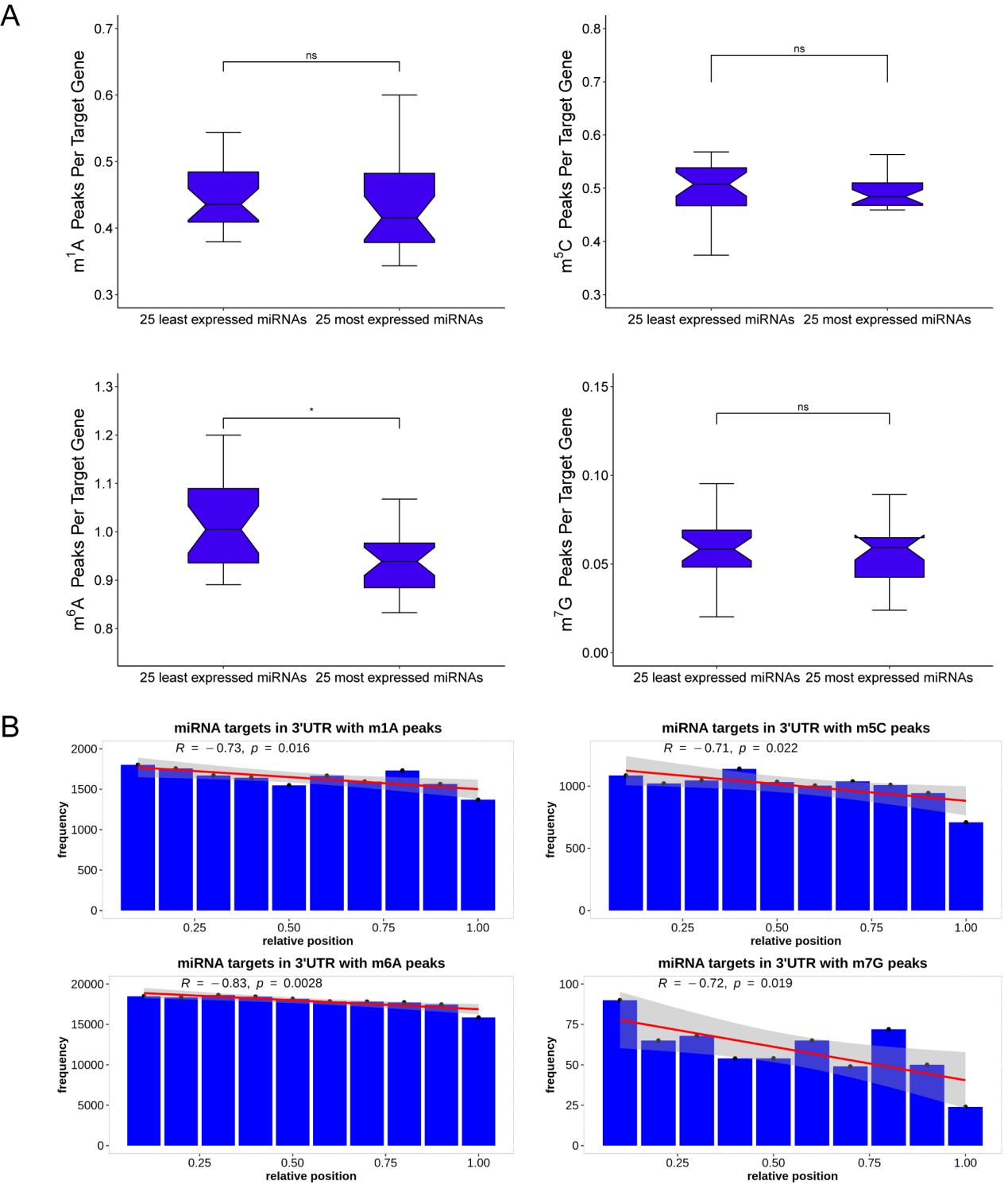
Supplemental Figure 4. The closest relationship between m6A and the miRNA-binding site in 3’UTRs**

**A.** Association between 3’UTR methylation abundance and miRNA targeting. Methylation abundance of the targeted 3’UTR for the 25 most/least abundant miRNAs in brain are shown using a box-plot, revealing a significantly greater percentage of m6A peaks within target mRNA 3'UTRs for the 25 weakly expressed brain miRNAs compared with the 25 most abundant brain miRNAs (*p < 0.05, Wilcoxon test). **B.** Distribution of miRNA target sites along the 3' UTRs of methylated genes. 3'UTRs are proportionally divided into ten parts, and the miRNA target abundance within each part is calculated and displayed using a bar chart. The relativity of miRNA target sites was calculated and displayed using the Pearson correlation coefficient.

**
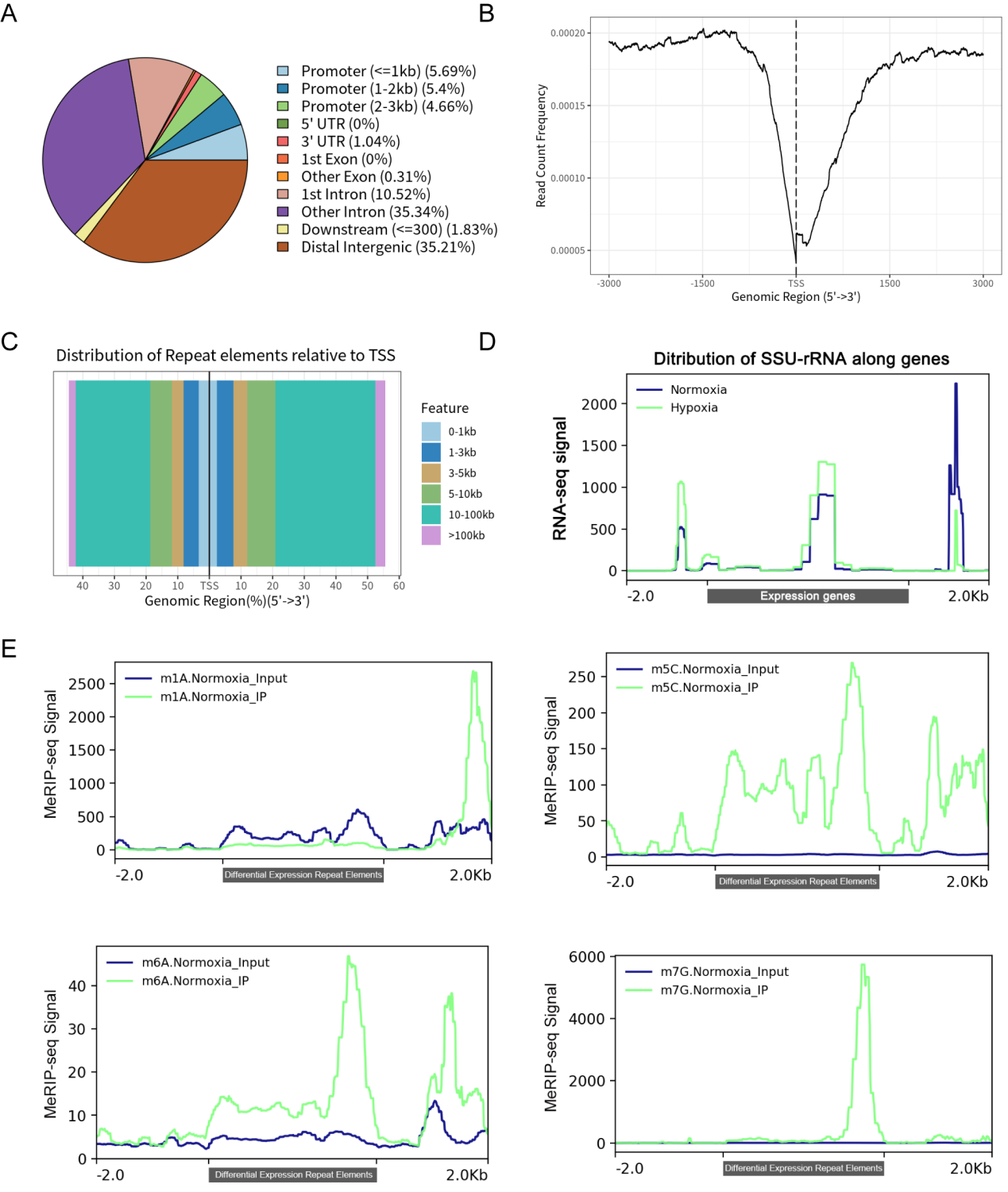
**

**Supplemental Figure 5. Description of the distribution of repeat elements in zebrafish brain tissue genomes**

**A-C**. Distribution of REs on the genome. The location information of REs was extracted from the featureCounts results, and ChIPseeker was used to analyze the distribution of these REs in the genome. **A**. The distribution of REs in each region of the genome. **B**. The distribution of REs in the range of 3 kb upstream and downstream of the TSS. **C**. The distribution percentage of REs within the scope of ±100 kb of the TSS. **D**. The distribution of SSU-rRNA on its corresponding genes. First, after extracting the location information of SSU-rRNA, bedtools was utilized to annotate those regions to the gene level, and the location information of its corresponding gene was obtained. Additionally all the SSU-rRNA sequences were extracted from the BAM file of RNA-seq, after which, deepTools was used to calculate and draw the SSU-rRNA distribution on these genes. **E**. The distribution of four methylation modification peaks on REs. To extract the location information of REs from the featureCounts results, deepTools was used to calculate the normalization matrix of MeRIP-seq and RE location information and to draw the distribution of methylation modification peaks on REs.
